# Dietary Patterns and Levels of Blood Pressure and Serum Lipids in a Japanese Population

**DOI:** 10.2188/jea.18.58

**Published:** 2008-04-10

**Authors:** Atsuko Sadakane, Akizumi Tsutsumi, Tadao Gotoh, Shizukiyo Ishikawa, Toshiyuki Ojima, Kazuomi Kario, Yosikazu Nakamura, Kazunori Kayaba

**Affiliations:** 1 Department of Public Health, Jichi Medical University.; 2 Occupational Training Center, University of Occupational and Environmental Health, Japan.; 3 Wara National Health Insurance Clinic.; 4 Division of Community and Family Medicine, Center for Community Medicine, Jichi Medical University.; 5 Department of Community Health and Preventive Medicine, Hamamatsu University School of Medicine.; 6 Division of Cardiology, Department of Internal Medicine, Jichi Medical University.; 7 Saitama Prefectural University, School of Health and Social Science.

**Keywords:** Diet, Factor Analysis, Statistical, Blood Pressure, Cholesterol, Japan

## Abstract

**Background:**

Associations between dietary patterns and cardiovascular disease risk factors remain unclear. The objective of this study was to evaluate the association between dietary patterns derived from factor analysis and the levels of blood pressure and serum lipids in a Japanese population.

**Methods:**

We conducted a cross-sectional analysis among 6886 (in the analysis on blood pressure) and 7641 (in the analysis on serum lipids) Japanese subjects aged 40-69 years. Dietary patterns were identified from a food frequency questionnaire by factor analysis. Associations between dietary patterns and blood pressure and serum lipids were examined after taking potential confounders into account.

**Results:**

Three dietary patterns were identified: vegetable, meat, and Western. In men, the meat pattern was associated with higher total, high-density lipoprotein (HDL), and low-density lipoprotein (LDL) cholesterol. The Western pattern was associated with higher total and LDL cholesterol. In women, the vegetable pattern was associated with lower systolic and diastolic blood pressure and pulse pressure, and higher HDL cholesterol. The meat pattern was associated with higher total and HDL cholesterol. The Western pattern was associated with higher total, HDL, and LDL cholesterol, and the least intake pattern of Western diet was associated with higher systolic and diastolic blood pressures.

**Conclusions:**

Dietary patterns of a Japanese population were related to cardiovascular disease risk factors, especially in women.

## INTRODUCTION

Diet is an important determinant of cardiovascular diseases. Several nutrients or foods are known to be risk or protective factors for cardiovascular diseases.^1^ With traditional nutrient studies using single nutrients or food approaches, there have been limitations in demonstrating the impact on disease outcomes because of difficulties in explaining interactions between nutrients and in detecting small effects from single nutrients.^2^ Nutrients and foods are consumed in combination; therefore, to examine their effects on health outcomes separately is difficult and complicated. To provide comprehensive dietary indices, dietary pattern analysis, which considers how foods are consumed in combination, has emerged as a possible approach to examine diet-disease relations.^3^ Among recent studies adopting this approach, dietary patterns have been defined from food frequency questionnaires (FFQs) using factor analysis. The relationships between dietary patterns and coronary heart disease, stroke, and diabetes have been investigated.^4^^-^^6^ Recently, a Japanese dietary pattern characterized by higher intake of soybean products, fish, seaweeds, vegetables, fruits, and green tea was found to be associated with lower risk of cardiovascular disease mortality.^7^

Several studies among Western populations have evaluated the associations between dietary patterns and risk factors for cardiovascular diseases, such as serum lipids, thrombogenic factors, glycemic indicators, inflammatory factors, and blood pressure.^8^^-^^10^ However, the associations reported between dietary patterns and serum lipids have been inconsistent. As well, investigations of associations between dietary patterns extracted by factor analysis and biological cardiovascular risk factors have seldom been done in non-Western populations. Among the few studies conducted in Japan, Mizoue et al,^11^ through a cross-sectional study, investigated the association between dietary patterns and glucose tolerance status among middle-aged men and found an inverse association between a diet characterized by higher intake of dairy products, fruits, vegetable and starch, and lower alcohol consumption and glucose intolerance.

In Japan, the incidence of cardiovascular diseases differs from that in Western countries, possibly partly the result of the traditional Japanese diet.^7^^,^^12^^,^^13^ Identifying dietary patterns and elucidating their relationships to cardiovascular risk factors, including blood pressure and serum lipids, among the Japanese population may offer important information about the prevention of cardiovascular diseases. The purpose of this study was to evaluate the association between dietary patterns, extracted by factor analysis, and the major cardiovascular risk factors, such as blood pressure and serum lipids, through a cross-sectional study among a Japanese population.

## METHODS

### Study Population

The Jichi Medical School Cohort study is a multicenter study exploring the risk factors for cardiovascular disease in Japanese subjects. Data were collected between 1992 and 1995. Ultimately, 12,490 Japanese individuals from 12 rural communities located across Japan participated in the study.^14^ In accordance with the provisions of the Health and Medical Service Law for the Aged, a mass screening program concerned with cardiovascular risk factors has been conducted in Japan since 1982. The law requires municipal governments to efficiently manage a program that is offered to all residents willing to participate. The target subjects vary between communities, from all residents to those not offered physical examinations at their workplace or elsewhere, including subjects of the National Health Insurance program. Residents aged 40-69 years were the subjects of the mass screening program in 8 of the 12 communities studied here, those aged 20-69 years were the subjects in one program, those aged ≥30 years were included in another, and all adult residents were included in the remaining two. In each community, the local government office invited all potential participants by sending letters or using public information sources. The invitation mentioned that persons visiting hospitals or clinics because of cardiovascular diseases did not have to take the examination. People other than those in the initially defined age groups also participated in the study, and are included in the database. The overall response rate was 65.4%. 12,490 subjects (4911 men and 7579 women) were eligible for all ages (19-93 years). Participants aged 40-69 years, answered all the questions regarding dietary intake, and whose blood pressure and serum lipids level were properly measured were subjected to the analyses. To avoid the effects of treatment of related diseases, we excluded subjects who received treatment for hypertension in the analysis of blood pressure or for dyslipidemia in the analysis of serum lipids. Consequently, 6886 (2742 men and 4144 women, for the analysis of blood pressure) and 7641 (2992 men and 4649 women, for the analysis of serum lipids) subjects were analyzed.

### Procedures

Sociodemographic and behavioral variables were ascertained by a standardized questionnaire, which included information on age, marital status, education, smoking status, alcohol consumption, and physical activity. The questionnaire was given to the subjects beforehand to complete on their own. The study design and procedures were reviewed and approved by each municipal government and the Ethics Committee for Epidemiological Research at Jichi Medical School. Written informed consent was obtained from all prospective participants.

### Sociodemographic and Behavioral Profiles

Marital status was coded as currently married or unmarried. Educational attainment was categorized into two strata: lower or higher than the level of compulsory education. Smoking habits were classified as never smoked, ex-smoker, 1-20 cigarettes per day, or ≥21 cigarettes per day for men, and never smoked, ex-smoker or current smoker for women. The total average amount of alcohol consumed in one typical drinking session was calculated, after taking into account the amount and alcohol content in specific beverages. Alcohol intake was categorized into non-drinker, <1 *go* (*go* is a traditional Japanese alcohol unit equivalent to 22.8 g alcohol), 1-3 *go* (22.8-68.2 g alcohol), or >3 *go* (>68.3 g alcohol) for men, and non-drinker, <1 *go* (<22.8 g alcohol), or ≥1 *go* (≥22.8 g alcohol) for women. The physical activity index, which was developed in the Framingham Study,^15^ was calculated by totaling the hours at each level of activity within a day, and multiplying this by a weight based on the oxygen consumption required for that activity. The index was categorized into three strata: low (≤28.4), medium (28.5-36.4), or high (≥36.5).

### Dietary Patterns

The usual dietary intakes of the participants were ascertained by employing an FFQ, composed of the 30 different foods most likely to be consumed. The FFQ was created based on that used in the Japan Collaborative Cohort (JACC) Study that demonstrated acceptable reproducibility and validity.^16^ By using the FFQ, participants’ frequencies of intake for each food were assessed by five-level scale questions: 1: seldom, 2: 1-2 times per month, 3: 1-2 times per week, 4: 3-4 times per week, and 5: almost every day. The items were subjected to a principal component analysis with varimax rotation. Nine factors were with eigenvalues >1.0 extracted. Of the derived factors, three interpretable factors were retained based on the Scree test; these were labeled as vegetable, meat, and Western patterns. These factors accounted for 28.5% of the variance explained. Almost the same factors were derived from principal component analyses by sex. Cronbach’s alpha coefficient for each dietary pattern indicated modest internal validity of these measures (0.76 for vegetable, 0.60 for meat, and 0.55 for Western patterns). For each participant, we calculated a vegetable pattern score, a meat pattern score, and a Western pattern score by summing the frequency of intake score of foods (five-point), weighted by the factor loadings of the foods. Participants were grouped into one of four linear strata based on the quartile of factor scores, separately for men and women.

### Physical Checkups

Physical examinations took place in each community. Body height was measured without shoes. Body weight was recorded with the subject clothed, and 0.5 kg in summer or 1 kg in other seasons was subtracted from the recorded weight. Body mass index (BMI) was categorized into tertiles based on the total sample distribution (<21.6 kg/m^2^, 21.6-23.9 kg/m^2^, or ≥24.0 kg/m^2^). Systolic and diastolic blood pressures were measured with a fully automated sphygmomanometer, BP203RV-II (Nippon Colin, Komaki, Japan), placed on the right arm of the subject, after he/she had been sitting for 5 min.

Blood samples were drawn from the antecubital vein of seated subjects, with minimal tourniquet use. Specimens were collected in siliconized vacuum glass tubes containing no additives. Tubes were centrifuged at 3000 ***g*** for 15 min at room temperature. After separation, serum samples were stored in refrigerated containers with dry ice for a maximum of 6 hrs, and then frozen as rapidly as possible to -80°C for storage until laboratory determinations were performed. Total cholesterol and triglycerides were measured by an enzymatic method (Wako, Osaka, Japan). High-density lipoprotein (HDL) cholesterol was measured by the phosphotungstate precipitation method (Wako, Osaka, Japan). Low-density lipoprotein (LDL) cholesterol levels were calculated by Friedwald’s equation.^17^ Blood variables were measured at the central laboratory of the Special Reference Laboratory (SRL; Tokyo, Japan), a commercial hematology laboratory.

### Statistical Analyses

All analyses were performed separately for women and men. First, associations between dietary patterns and sociodemographic and lifestyle profiles were examined by chi-square test. Then, analysis of covariance was performed, in which physical parameters such as systolic and diastolic blood pressure, pulse pressure, and total, HDL, and LDL cholesterol levels were defined as dependent variables, and each dietary pattern was defined as an independent variable. Study area, age, marital status, education level, smoking status, alcohol consumption, physical activity index, and BMI were selected as potential confounding factors. Total energy intake calculated according to the JACC study method,^16^ was also included as a confounder. Energy contained in each food was based on the Standard Tables of Food Composition in Japan, 5th revised edition.^18^ Linear trends for the associations were assessed by computing the *P* value for each trend with multiple regression analysis. All tests were two-tailed and *P*<0.05 was considered statistically significant. Statistical analysis was performed using SPSS^®^ for Windows, ver. 11.5.

## RESULTS

General characteristics of the study subjects are shown in Table 1.

**Table 1.** General characteristics of study subjects.

	Men		Women	
Age (%)		(3148)		(4909)
40-49 years	26.3		23.4	
50-59	28.0		33.0	
60-64	24.9		22.6	
65-69	20.9		21.0	
Mean age (years) ^*^	56.3 ± 8.7		56.6 ± 8.4	

Marital status (%)		(3126)		(4885)
Married	94.4		92.9	
Unmarried	5.6		7.1	

Education level (age at completion, %)		(3121)		(4874)
-15 years	46.1		53.7	
16+ years	53.9		46.3	

Smoking status (%)		(3135)		(4828)
Non smoker	22.0		93.4	
Ex-smoker	28.4		1.9	
Current smoker, -20 /day	35.8		4.7 ^†^	
Current smoker, 20+ (Men)	13.9		—	

Alcohol consumption (%)		(3046)		(4671)
Non-drinker	24.5		75.8	
-22.7 g	22.6		19.3	
22.8-68.2	41.5		4.9 ^‡^	
68.3+ (Men)	11.4		—	

Physical activity index (%) ^§^		(3132)		(4883)
-28.4	21.4		25.5	
28.5-36.4	40.2		57.0	
36.5+	38.4		17.5	

Body mass index (kg/m^2^) ^*^	23.1 ± 2.9	(3088)	23.3 ± 3.1	(4838)
Treatment of hypertension (%)	9.9	(3088)	13.0	(4813)
Treatment of dyslipidemia (%)	1.3	(3065)	2.2	(4790)
Systolic blood pressure (mmHg) ^*^	132.0 ± 20.5	(3105)	129.3 ± 20.8	(4852)
Diastolic blood pressure (mmHg) ^*^	79.8 ± 12.3	(3105)	76.9 ± 11.9	(4852)
Pulse pressure (mmHg) ^*^	52.2 ± 11.6	(3105)	52.4 ± 12.4	(4852)

Total cholesterol (mg/dl) ^*^	186.0 ± 34.3	(3118)	199.2 ± 33.7	(4871)
HDL cholesterol (mg/dl) ^*^	48.7 ± 13.4	(3119)	52.5 ± 12.4	(4871)
LDL cholesterol (mg/dl) ^*^	111.5 ± 31.8	(3118)	124.3 ± 31.0	(4870)

* : Mean ± standard deviation.

† : Those women who smoked cigarettes were grouped as current smokers irrespective of the number of cigarettes smoked per day.

‡ : Alcohol consumption was categorized into non-drinker, <22.8 g and ≥22.8 g for women.

§ : Physical activity index refers to metabolic equivalent task hours.

The number of subjects observed for each item in parentheses

HDL cholesterol: high-density lipoprotein cholesterol

LDL cholesterol: low-density lipoprotein cholesterol

Results of the factor analyses are shown in Table 2. The vegetable pattern was characterized by higher intakes of vegetables, potatoes, soybeans products tofu and fermented soybeans, fruits, sea weeds, citrus, beans, and dried fish (according to the magnitude of factor loadings). The meat pattern was characterized by higher intakes of processed meats, beef, pork, poultry, steamed fish paste, high-fat products, and butter. The Western pattern was characterized by higher intakes of breads, butter, and yoghurt, and lower intakes of rice, salty products, and miso soup.

**Table 2.** Factor-loading matrix for the three dietary patterns identified from the food frequency questionnaire.

Foods	Vegetable	Meat	Western
Yellow and orange vegetables	**0.67**	-0.05	0.09
Other vegetables	**0.63**	-0.03	0.05
Potatoes	**0.59**	-0.06	0.00
Fruits	**0.55**	0.00	0.33
Tofu/fermented soybeans	**0.56**	-0.01	0.00
Sea weeds	**0.54**	0.07	0.11
Citrus^*^	**0.51**	0.04	0.29
Beans	**0.47**	0.12	0.05
Dried fish	**0.46**	0.21	-0.16
Processed meats	0.14	**0.62**	0.01
Pork	0.31	**0.52**	-0.03
Beef	-0.07	**0.54**	0.23
Poultry	0.26	**0.48**	0.03
Steamed fish paste	0.30	**0.45**	-0.02
High-fat products ^†^	-0.10	**0.41**	-0.32
Bread	0.01	0.28	**0.59**
Butter	0.13	**0.40**	**0.53**
Rice	-0.13	0.09	**-0.51**
Salty products ^‡^	-0.12	0.26	**-0.48**
Miso-soup	0.33	-0.15	**-0.43**
Yoghurt	0.26	0.10	**0.42**
Fish	0.35	0.21	0.07
Eggs	0.31	0.28	0.01
Snacks	0.28	0.23	0.04
Noodles	0.24	0.18	0.11
Coffee	-0.21	0.38	0.23
Juice	-0.10	0.34	-0.12
Milk	0.25	0.04	0.38
Green tea	0.08	0.06	-0.10
Pickles	0.32	0.13	-0.31
Variance explained (%)	13.0	8.1	7.4

Loadings with an absolute value more than 0.40 are shown in bold.

* : oranges, lemons, and grapefruit

† : taste for fatty foods

‡ : taste for salty foods

Sociodemographic and lifestyle characteristics were associated with dietary patterns, as shown in Table 3. In men, those who had a higher vegetable pattern score were older, married, and less likely to smoke. Men with the least intake of vegetables drank heavily. Those who had a higher meat pattern score were younger, married, highly educated, and likely to smoke and drink alcohol. Those who had a higher Western pattern score were younger, highly educated, less likely to smoke and drink alcohol, but were physically inactive. In women, those who had a higher vegetable pattern score were older, married, highly educated, less likely to smoke and drink alcohol. Those who had a higher meat pattern score were younger, married, highly educated, and likely to drink alcohol. Those who had a higher Western pattern score were younger, unmarried, highly educated, likely to drink alcohol, and physically inactive.

**Table 3.** Sociodemographic and lifestyle categories by quartiles of dietary pattern scores in men and women.

	Vegetable pattern					Meat pattern					Western pattern				
		
	Q1^*^	Q2	Q3	Q4	*P*	Q1	Q2	Q3	Q4	*P*	Q1	Q2	Q3	Q4	*P*
	Men (%)														
Age															
40-49 years	35	27	25	18	<0.001	18	26	30	32	<0.001	23	24	29	30	0.012
50-59	27	28	29	28		27	30	27	28		31	29	25	27	
60-64	23	24	26	27		30	26	22	21		27	26	24	23	
65-69	15	21	20	28		24	18	22	19		19	21	22	21	

Marital status															
Married	91	95	96	96	<0.001	91	94	96	97	<0.001	93	95	94	96	0.078
Unmarried	9	6	4	4		9	6	4	3		7	5	6	4	

Education level ^†^															
16+ years	54	53	56	53	0.735	45	54	58	59	<0.001	46	50	57	63	<0.001
-15	46	47	44	47		55	46	43	41		54	50	44	37	

Smoking status															
Non smoker	18	20	24	26	<0.001	24	23	20	22	0.007	20	20	25	23	0.031
Ex-smoker	24	30	31	28		29	27	32	25		26	29	28	31	
Current smoker, -20/day	39	36	33	35		37	36	33	38		39	36	35	33	
Current smoker, 21+	19	14	11	11		10	14	15	15		16	14	13	12	

Alcohol consumption															
Non-drinker	25	25	23	25	0.011	28	23	24	23	0.027	21	25	24	28	<0.001
-22.7 g	22	21	26	22		19	22	22	27		16	23	26	26	
22.8-68.2	38	44	41	43		40	43	42	41		49	41	41	36	
68.3+	15	10	10	10		12	12	11	10		15	11	9	11	

Physical activity index ^‡^															
-28.4	23	23	19	21	0.540	22	22	21	21	0.218	16	19	21	30	<0.001
28.5-36.4	39	39	42	41		40	42	42	36		37	40	41	42	
36.5+	38	38	39	38		38	36	37	42		47	41	38	28	

Body mass index															
-21.5 kg/m^2^	32	32	32	34	0.514	32	32	32	35	0.369	30	35	34	31	0.158
21.6-23.9	33	36	34	32		32	37	34	32		33	32	34	36	
24.0+	36	32	34	33		36	32	34	33		37	33	32	32	

	Women (%)														
Age															
40-49 years	26	26	23	19	0.005	12	23	25	35	<0.001	16	19	26	33	<0.001
50-59	32	32	34	35		33	33	34	33		33	32	34	33	
60-64	21	22	23	24		26	23	23	18		27	25	21	18	
65-69	21	21	20	22		29	22	19	14		24	24	19	17	

Marital status															
Married	90	93	93	95	<0.001	90	93	93	95	<0.001	93	93	94	91	0.005
Unmarried	10	7	7	5		10	7	7	5		7	7	6	9	

Education level ^†^															
16+ years	39	46	50	50	<0.001	32	45	50	59	<0.001	30	41	50	64	<0.001
-15	61	54	50	50		68	56	50	41		70	59	50	36	

Smoking status															
Non smoker	90	93	95	96	<0.001	93	94	94	93	0.691	94	92	94	94	0.058
Ex-smoker	2	2	2	2		2	2	2	2		1	2	2	2	
Current smoker	8	4	4	3		6	4	5	5		5	6	4	4	

Alcohol consumption															
Non drinker	71	75	78	80	<0.001	81	76	74	72	<0.001	77	77	74	75	0.033
-22.7 g	22	19	19	17		14	19	22	22		17	18	21	21	
22.8+	7	6	4	3		5	5	4	6		5	5	5	4	

Physical activity index ^‡^															
-28.4	28	26	24	23	0.136	25	27	25	25	0.441	21	24	26	31	<0.001
28.5-36.4	55	56	58	59		59	55	56	58		56	58	57	58	
36.5+	16	18	17	18		17	18	19	17		23	18	17	12	

Body mass index															
-21.5 kg/m^2^	31	29	32	31	0.273	29	31	30	33	0.169	29	31	31	31	0.153
21.6-23.9	32	35	35	33		33	32	33	35		32	34	33	35	
24.0+	38	36	34	37		38	37	37	33		39	35	36	34	

* : Q1 is the lowest and Q4 is the highest quartile score for each dietary pattern.

† : Age at completion.

‡ : Physical activity index refers to metabolic equivalent task hours.

Table 4 shows the association between each dietary pattern and biological cardiovascular risk factors. In men, those who were in a higher quartile of the meat pattern had higher total, HDL, and LDL cholesterol (*P* for trend <0.05 for all). Those who were in a higher quartile of the Western pattern had higher total and LDL cholesterol (*P* for trend <0.05 for both). In women, those who were in a higher quartile of the vegetable pattern had lower systolic blood pressure, diastolic blood pressure, and pulse pressure, and also had higher HDL cholesterol (*P* for trend <0.05 for all). Those who were in a higher quartile of the meat pattern had higher total and HDL cholesterol (*P* for trend <0.05 for both). Those who were in the lowest quartile of the Western pattern had the highest systolic and diastolic blood pressure, and those who were in a higher quartile of the Western pattern had higher total, HDL, and LDL cholesterol (*P* for trend <0.05 for all cholesterol level).

**Table 4.** Mean biomarker values^*^ by quartiles of dietary pattern scores.

	Vegetable dietary pattern						Meat dietary pattern						Western dietary pattern					
		
	Q1 ^†^	Q2	Q3	Q4	*P* ^‡^	*P* ^§^	Q1	Q2	Q3	Q4	*P* ^‡^	*P* ^§^	Q1	Q2	Q3	Q4	*P* ^‡^	*P* ^§^
	Men																	
n ^¶^	657	655	644	630			609	652	649	678			630	644	640	665		
Systolic blood pressure (mmHg)	130.8 (129.3-132.3)^||^	129.6 (128.2-131.0)	129.9 (128.4-131.3)	130.7 (129.2-132.2)	0.586	0.979	130.4 (128.9-131.9)	130.6 (129.1-132.0)	130.1 (128.7-131.6)	129.8 (128.4-131.3)	0.905	0.526	130.5 (129.0-132.0)	130.1 (128.7-131.6)	130.0 (128.5-131.4)	130.5 (129.1-132.0)	0.937	0.981
Diastolic blood pressure (mmHg)	78.8 (77.9-79.7)	78.4 (77.6-79.3)	78.7 (77.8-79.6)	79.2 (78.3-80.1)	0.699	0.508	79.0 (78.1-80.0)	78.8 (78.0-79.7)	78.6 (77.7-79.4)	78.7 (77.8-79.5)	0.904	0.529	78.7 (77.8-79.6)	78.8 (77.9-79.6)	78.7 (77.8-79.5)	79.0 (78.1-79.9)	0.951	0.713
Pulse pressure (mmHg)	52.0 (51.1-52.9)	51.2 (50.3-52.0)	51.2 (50.3-52.0)	51.5 (50.6-52.4)	0.492	0.464	51.4 (50.5-52.3)	51.8 (50.9-52.6)	51.6 (50.7-52.4)	51.2 (50.3-52.0)	0.766	0.658	51.8 (50.9-52.7)	51.3 (50.5-52.2)	51.3 (50.5-52.2)	51.5 (50.7-52.4)	0.861	0.673
n	695	720	686	691			670	721	688	714			683	696	697	707		
Total cholesterol (mg/dl)	184.7 (182.1-187.2)	186.3 (183.9-188.8)	186.7 (184.2-189.2)	186.9 (184.3-189.5)	0.623	0.240	181.7 (179.1-184.3)	186.4 (184.0-188.8)	188.4 (186.0-190.9)	187.9 (185.4-190.4)	0.001	0.001	183.7 (181.2-186.3)	184.5 (182.0-186.9)	186.6 (184.1-189.0)	189.5 (187.0-192.0)	0.007	0.001
HDL cholesterol (mg/dl)	47.8 (46.8-48.7)	49.2 (48.3-50.1)	48.6 (47.7-49.5)	49.4 (48.4-50.3)	0.098	0.067	47.5 (46.6-48.5)	48.5 (47.6-49.4)	49.3 (48.4-50.2)	49.6 (48.7-50.6)	0.015	0.002	48.7 (47.8-49.7)	48.8 (47.9-49.7)	48.8 (47.9-49.7)	48.5 (47.6-49.4)	0.955	0.731
LDL cholesterol (mg/dl)	110.8 (108.4-113.1)	112.4 (110.2-114.6)	112.9 (110.6-115.1)	111.6 (109.3-113.9)	0.584	0.586	107.2 (104.9-109.5)	112.3 (110.1-114.5)	113.9 (111.6-116.1)	113.9 (111.7-116.2)	<0.001	<0.001	109.1 (106.8-111.4)	110.5 (108.3-112.8)	112.1 (109.9-114.4)	115.5 (113.2-117.7)	0.001	<0.001

	Women																	
n	969	969	975	931			896	955	984	1006			935	939	971	994		
Systolic blood pressure (mmHg)	128.7 (127.4-129.9)	126.9 (125.7-128.0)	125.4 (124.3-126.6)	124.1 (122.9-125.4)	<0.001	<0.001	127.1 (125.9-128.4)	126.3 (125.1-127.4)	126.2 (125.0-127.3)	125.7 (124.5-126.9)	0.501	0.149	128.0 (126.8-129.2)	125.6 (124.4-126.8)	125.7 (124.5-126.8)	126.0 (124.8-127.2)	0.016	0.036
Diastolic blood pressure (mmHg)	76.4 (75.7-77.1)	75.3 (74.6-76.0)	74.9 (74.2-75.6)	74.5 (73.7-75.2)	0.004	0.001	75.9 (75.2-76.7)	75.1 (74.4-75.8)	75.2 (74.6-75.9)	75.0 (74.2-75.7)	0.312	0.128	76.4 (75.7-77.1)	74.4 (73.7-75.1)	75.0 (74.3-75.7)	75.4 (74.7-76.1)	0.001	0.149
Pulse pressure (mmHg)	52.3 (51.5-53.0)	51.6 (50.9-52.3)	50.5 (49.8-51.2)	49.7 (48.9-50.4)	<0.001	<0.001	51.2 (50.4-52.0)	51.2 (50.5-51.9)	51.0 (50.3-51.7)	50.7 (50.0-51.5)	0.825	0.364	51.6 (50.9-52.3)	51.2 (50.5-51.9)	50.7 (50.0-51.3)	50.6 (49.9-51.3)	0.192	0.038
n	1069	1071	1079	1046			1026	1068	1087	1086			1048	1058	1076	1077		
Total cholesterol (mg/dl)	198.8 (196.8-200.9)	198.5 (196.6-200.4)	198.6 (196.7-200.5)	200.1 (198.0-202.2)	0.685	0.448	197.9 (195.8-199.9)	197.1 (195.2-199.1)	199.8 (197.9-201.8)	201.0 (199.0-203.1)	0.042	0.014	196.3 (194.3-198.3)	195.8 (193.9-197.8)	200.2 (198.3-202.1)	203.5 (201.5-205.4)	<0.001	<0.001
HDL cholesterol (mg/dl)	51.3 (50.6-52.1)	51.6 (50.9-52.3)	52.6 (51.9-53.3)	54.3 (53.6-55.1)	<0.001	<0.001	50.8 (50.0-51.6)	52.1 (51.3-52.8)	53.6 (52.9-54.3)	53.2 (52.5-54.0)	<0.001	<0.001	51.0 (50.2-51.7)	51.6 (50.8-52.3)	53.0 (52.3-53.7)	54.3 (53.5-55.0)	<0.001	<0.001
LDL cholesterol (mg/dl)	125.3 (123.5-127.2)	124.8 (123.1-126.6)	124.3 (122.5-126.1)	123.7 (121.8-125.7)	0.727	0.252	124.4 (122.5-126.4)	123.5 (121.7-125.3)	124.5 (122.7-126.2)	125.7 (123.9-127.6)	0.425	0.287	122.5 (120.7-124.3)	122.6 (120.8-124.3)	125.1 (123.3-126.8)	127.9 (126.1-129.7)	<0.001	<0.001

* : Adjusted for study area, age, marital status, education level, smoking status, alcohol consumption, physical activity index, total energy intake, and body mass index.

† : Q1 is the lowest, Q4 is the highest quartile for score of each dietary pattern.

‡ : *P* value computed by analysis of covariance.

§ : *P* for trend computed by multiple regression analysis.

|| : Mean (95% CI).

¶ : Number of subjects varied between dietary patterns because missing value arose when we adjusted confounding factors and rounded off factor scores of each dietary pattern.

HDL cholesterol: high-density lipoprotein cholesterol

LDL cholesterol: low-density lipoprotein cholesterol

## DISCUSSION

We identified three dietary patterns, vegetable, meat and Western, by factor analysis using data collected using FFQs. The vegetable pattern was a diet rich in vegetables, fruit, and Japanese traditional foods, whereas the meat and Western patterns contained foods such as meat, dairy products and bread, all of which were introduced and have become popular in Japan along with the spread of Western culture. The vegetable pattern was related to lower systolic and diastolic blood pressure and pulse pressure, and higher HDL cholesterol in women. The meat pattern was related to higher total, HDL, and LDL (only in men) cholesterol. The lowest Western pattern was related to the highest systolic and diastolic blood pressure in women, and the Western pattern was related to higher total, HDL (only in women) and LDL cholesterol. These results were observed independent of possible confounding factors such as study area, age, marital status, education level, smoking status, alcohol consumption, physical activity, total energy intake, and BMI.

In our study, women presented more significant associations between dietary patterns and cardiovascular disease risks than did men. Although in general, energy intake is likely to be under-reported by women,^19^ measurements of food intake in our population might have been more accurate in women than in men. Among women, the mean total energy intake computed by FFQ and the Standard Tables of Food Composition was more closely related to that of the general Japanese population shown in The National Health and Nutrition Survey,^20^ than it was among men. A large measurement error could have reduced the power to detect the fine differences among men. Sex differences in the effect of dietary patterns on cardiovascular disease risk have been shown to depend on sex differences in cell metabolism and variations in confounders such as obesity and other lifestyle factors.^21^ Because smoking status was greatly different between men and women, we conducted a stratified analysis by smoking status. This additional analysis revealed that almost all significant associations disappeared among men, whereas significant associations remained among female non-smokers (the majority of women), suggesting that not only combination of foods but also combinations of dietary pattern and other lifestyle factors determine cardiovascular risk factors.

We found that the difference in systolic blood pressure from the top to bottom quartiles of the vegetable pattern in women was 4.6 mmHg. This difference seems clinically relevant because even a 2 mmHg lower than usual systolic blood pressure involves 10% lower stroke mortality and 7% lower mortality from ischemic heart disease or other vascular diseases in middle age.^22^ Pulse pressure has been recognized as a significant predictor of cardiovascular diseases in middle-aged and older people.^23^^,^^24^ An increase in pulse pressure of 10 mmHg has been reported to contribute to a 22% increase in cardiovascular mortality,^24^ and we found that the difference in pulse pressure from the top to bottom quartiles of the vegetable pattern in women was 2.6 mmHg, which may have just a modest clinical importance. The meat and Western dietary patterns were associated with high total and LDL cholesterol levels, with an adjusted difference from the top to bottom quartiles of 3-7 mg/dL for total and 5-6 mg/dL for LDL cholesterol. Because lipid lowering intervention studies have suggested that substantial reductions in lipid levels are associated with significant reductions in cardiovascular diseases,^25^^-^^27^ the magnitude of the observed effect might not be clinically meaningful. As well, the meat and Western patterns were also associated with higher HDL cholesterol, which is recognized as a protective factor for cardiovascular diseases. This suggests that the impact of these dietary patterns on cardiovascular disease incidence may not be clinically meaningful.

Previous studies have shown inconsistent associations between dietary patterns and serum lipids. In the Monitoring Project on Risk Factors and Chronic Diseases (MORGEN study) among a Dutch population, traditional (high intake of red meat and potatoes, and low intake of low-fat dairy products and fruits) and refined (high intake of French fries, high-sugar beverages, and white bread, and low intake of whole-grain bread and boiled vegetables) food patterns were associated with higher total cholesterol.^9^ Whereas, American studies produced negative findings. Western dietary patterns characterized by high intake of red meat, processed meat, high-fat foods and refined grains were not associated with total plasma cholesterol, in the Health Professionals Follow-up Study.^8^ A similar association was found in the NHANES III study, in which a Western dietary pattern characterized by high intake of processed meats, eggs, red meat and high-fat dairy products was not associated with serum total cholesterol.^10^

With regard to blood pressure, in the MORGEN study, traditional and refined food patterns were associated with higher blood pressure, and cosmopolitan patterns (high intake of fried vegetables, salad, rice, chicken, fish and wine) were associated with lower systolic blood pressure.^9^ However, many cross-sectional^21^^,^^28^ and prospective^29^ studies have failed to show significant associations between dietary patterns and blood pressure. In the Dietary Approaches to Stop Hypertension (DASH) study that investigated the effect of a diet rich in fruits, vegetables, low-fat dairy products with reduced saturated and total fat (the DASH diet) on blood pressure; the diet was shown to reduce systolic blood pressure by 5 mmHg.^30^ An almost comparable level of difference in systolic blood pressure was found from the top to bottom quartiles for the vegetable pattern in our female population. Besides the slightly different dietary contents between the DASH diet and our vegetable pattern, the relatively higher salt consumption of the Japanese population might explain the lower level of blood pressure differences. The salt consumption of the average Japanese person is estimated at 10.5-12.0 g/day,^20^ whereas that in the DASH trial was 7.6 g/day.^30^

Operationalization of dietary patterns extracted from the FFQ by using factor analysis needs to be discussed. With respect to the internal consistency of dietary patterns, Cronbach’s alpha coefficient of each dietary pattern in the present study was not so high, but was acceptable. Nevertheless, the associations between dietary patterns and biological cardiovascular risk factors were shown to be significant. Similar dietary patterns to the vegetable and meat patterns in our study have been identified among another Japanese populations.^7^^,^^11^^,^^31^^,^^32^ This suggests that the dietary pattern approach using factor analysis is reproducible. In other studies, dietary patterns extracted by factor analysis have been shown to remain consistent for long periods of time.^8^^,^^33^^,^^34^ Reasonable measurement validity has been demonstrated by comparing two sets of dietary patterns extracted from FFQs and actual diet records.^33^ In our study, observed associations between dietary patterns and both sociodemographic and lifestyle characteristics were consistent with those in other studies; showing that healthy dietary patterns are associated with health-related characteristics and behavior.^35^^-^^37^ However, because our FFQ was not validated, potential measurement errors may have affected the results.

There were some limitations in our study. First, the study subjects may not be representative of the whole Japanese population. Subjects were recruited through government-promoted health checkups, in which they participated voluntarily, and this may have realized a population with a greater concern for health. Moreover, of the total registered subjects, only 60%, which were not receiving treatment for hypertension or dyslipidemia were subjected to the analyses. Further investigations among representative populations will be necessary to better ascertain the association between dietary patterns and cardiovascular disease risk factors. Second, awareness of previously diagnosed hypertension or dyslipidemia can alter dietary patterns. Because of the cross-sectional study, the causal relationship between dietary patterns and cardiovascular disease risk could not be determined, and the possibility of reverse causality may have arisen. In a Spanish population, awareness of having hypertension was related to an increase in the consumption of vegetables.^38^ Although we excluded subjects who had received treatment, there might have been subjects who knew their physical status but did not receive actual medical treatment. Despite these limitations, our study had several strengths. It was a community-based, large-scale study dealing with dietary patterns and biological cardiovascular risk factors in both men and women. Furthermore, it was highly standardized in how it obtained sociodemographic and lifestyle characteristics as well as biological cardiovascular risk factors.

In conclusion, our study indicated that dietary patterns were related to major biological cardiovascular risk factors such as blood pressure and serum lipids, especially in women. In particular, the vegetable pattern was significantly associated with favorable cardiovascular disease risk factors, lower blood pressure and higher HDL cholesterol in women. The association between the meat and Western patterns and serum lipids was statistically significant but modest, thus these patterns may have less clinical significance. Japanese vegetable-rich diet may reduce cardiovascular disease risk through lowering blood pressure.

## APPENDIX

This study was financially supported by a Scientific Research Grant from the Ministry of Education, Culture, Sports, Science and Technology, Japan, and by grants from the Foundation for the Development of the Community, Tochigi, Japan.

The Jichi Medical School Cohort Study Group: Akizumi Tsutsumi (University of Occupational and Environmental Health), Atsuko Sadakane (Jichi Medical University), Atsushi Hashimoto (Aichi Prefectural Aichi Hospital), Eiji Kajii (Jichi Medical University), Hideki Miyamoto (former Jichi Medical School), Hidetaka Akiyoshi (Fukuoka University School of Medicine), Hiroshi Yanagawa (Jichi Medical University), Hitoshi Matsuo (Gifu Prefectural Hospital), Jun Hiraoka (Tako Central Hospital), Kaname Tsutsumi (Kyushu International University), Kazunori Kayaba (Saitama Prefectual University), Kazuomi Kario (Jichi Medical University), Kazuyuki Shimada (Jichi Medical University), Kenichiro Sakai (Akaike Town Hospital), Kishio Turuda (Takasu National Health Insurance Clinic), Machi Sawada (Agawa Osaki National Health Insurance Clinic), Makoto Furuse (Jichi Medical University), Manabu Yoshimura (Kuze Clinic), Masahiko Hosoe (Gero Hot-Spring Hospital), Masahiro Igarashi (Igarashi Clinic), Masafumi Mizooka (Kamagari National Health Insurance Clinic), Naoki Nago (Yokosuka General Hospital Uwamachi), Nobuya Kodama (Sakugi Clinic), Noriko Hayashida (Tako Central Hospital), Rika Yamaoka (Awaji-Hokudan Public Clinic), Seishi Yamada (Wara National Health Insurance Hospital), Shinichi Muramatsu (Jichi Medical University), Shinya Hayasaka (Jichi Medical University), Shizukiyo Ishikawa (Jichi Medical University), Shuzo Takuma (Akaike Town Hospital), Tadao Gotoh (Wara National Health Insurance Hospital), Takafumi Natsume (Oyama Municipal Hospital), Takashi Yamada (Kuze Clinic), Takeshi Miyamoto (former Okawa Komatsu National Health Insurance Clinic), Tomohiro Deguchi (Akaike Town Hospital), Tomohiro Saegusa (Sakuma National Health Insurance Hospital), Yoshihiro Shibano (Saiseikai Iwaizumi Hospital) Yoshihisa Ito (Asahikawa Medical College), and Yosikazu Nakamura (Jichi Medical University).
